# Exploring Clinical and Epidemiological Characteristics of Interstitial Lung Diseases: Rationale, Aims, and Design of a Nationwide Prospective Registry—The EXCITING-ILD Registry

**DOI:** 10.1155/2015/123876

**Published:** 2015-11-10

**Authors:** Michael Kreuter, Felix J. F. Herth, Margarethe Wacker, Reiner Leidl, Andreas Hellmann, Michael Pfeifer, Jürgen Behr, Sabine Witt, Dagmar Kauschka, Marcus Mall, Andreas Günther, Philipp Markart

**Affiliations:** ^1^Pneumology and Respiratory Critical Care Medicine, Centre for Interstitial and Rare Lung Diseases, Thoraxklinik, University of Heidelberg, 69126 Heidelberg, Germany; ^2^Translational Lung Research Centre Heidelberg (TLRCH), Member of the German Centre for Lung Research (DZL), 69120 Heidelberg, Germany; ^3^Institute of Health Economics and Healthcare Management, Helmholtz Centre Munich GmbH, German Research Centre for Environmental Health, Member of the German Centre for Lung Research (DZL), Comprehensive Pneumology Centre Munich (CPCM), 85764 Neuherberg, Germany; ^4^Institute of Health Economics and Healthcare Management, Munich Centre of Health Sciences, Ludwig Maximilian University of Munich, 80336 Munich, Germany; ^5^Association of German Pneumologists (BdP), 86150 Augsburg, Germany; ^6^Medical Clinic II, University of Regensburg and Klinikum Donaustauf, 93093 Donaustauf, Germany; ^7^Institute of Lung Research, 10405 Berlin, Germany; ^8^Medical Clinic V, University Clinic, Ludwig-Maximilians-University Munich, Comprehensive Pneumology Centre Munich (CPC-M), Member of the German Centre for Lung Research (DZL), 81377 München, Germany; ^9^Patient Support Group Lungenfibrose e.V., 45242 Essen, Germany; ^10^Department of Translational Pulmonology, University of Heidelberg, 69120 Heidelberg, Germany; ^11^Division of Paediatric Pulmonology & Allergy and Cystic Fibrosis Center, Department of Paediatrics III, University of Heidelberg, 69120 Heidelberg, Germany; ^12^Medical Clinic II, University Hospital Giessen, Universities of Giessen and Marburg Lung Centre (UGMLC), Member of the German Centre for Lung Research (DZL), 35392 Giessen, Germany; ^13^Medical Clinic V (Pneumology), Cardiothoracic Centre, Campus Fulda, University Medicine Marburg, 36043 Fulda, Germany

## Abstract

Despite a number of prospective registries conducted in past years, the current epidemiology of interstitial lung diseases (ILD) is still not well defined, particularly regarding the prevalence and incidence, their management, healthcare utilisation needs, and healthcare-associated costs. To address these issues in Germany, a new prospective ILD registry, “Exploring Clinical and Epidemiological Characteristics of Interstitial Lung Diseases” (EXCITING-ILD), is being conducted by the German Centre for Lung Research in association with ambulatory, inpatient, scientific pulmonology organisations and patient support groups. This multicentre, noninterventional, prospective, and observational ILD registry aims to collect comprehensive and validated data from all healthcare institutions on the incidence, prevalence, characteristics, management, and outcomes regarding all ILD presentations in the real-world setting. Specifically, this registry will collect demographic data, disease-related data such as ILD subtype, treatments, diagnostic procedures (e.g., HRCT, surgical lung biopsy), risk factors (e.g., familial ILD), significant comorbidities, ILD managements, and disease outcomes as well as healthcare resource consumption. The EXCITING-ILD registry will include in-patient and out-patient ILD healthcare facilities in more than 100 sites. In summary, this registry will document comprehensive and current epidemiological data as well as important health economic data for ILDs in Germany.

## 1. Introduction

**Figure 1:**
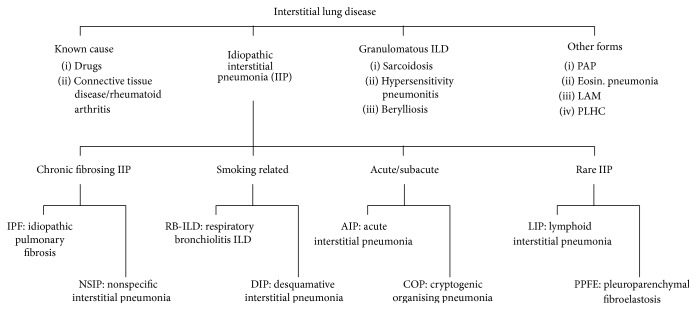
Classification of ILDs. PAP: pulmonary alveolar proteinosis, LAM: lymphangioleiomyomatosis, and PLHC: pulmonary Langerhans cell histiocytosis. Adapted from the American Thoracic Society, European Respiratory Society International Multidisciplinary Consensus Classification of the Idiopathic Interstitial Pneumonias, Am J Respir Crit Care Med. 2002, and Travis et al. Am J Respir Crit Care Med. 2013 [2, 3].

Interstitial (or diffuse parenchymal) lung diseases (ILDs) represent a large, heterogeneous group of more than 200 different entities, most of which are classified as rare diseases [1–3]. They are defined as lung diseases that affect the alveolar structures, the pulmonary interstitium, and small airways. A diagnosis of an ILD relies mainly on the combination of clinical, radiological, and pathological criteria, which should be explored in a multidisciplinary board. Among the ILDs, the most important are sarcoidosis, idiopathic pulmonary fibrosis (IPF), hypersensitivity pneumonitis (HP), also called extrinsic allergic alveolitis (EAA), ILD as a manifestation of connective tissue disease (CTD), and drug-induced ILD and pneumoconiosis [2, 3]. The majority of ILDs are idiopathic and include the group of idiopathic interstitial pneumonias (IIPs) (Figure 1). Only about one in three cases of ILD have an identifiable cause [1].

Despite being rare diseases, the recent Global Burden of Disease Study reported that interstitial lung diseases rank in 40th position of all diseases regarding global years of life lost in 2013, which represents an increase of 86% compared to 1990 [4]. The most common ILD, IPF, has a poor prognosis with median survival of 2-3 years from diagnosis [5]; for other forms of ILD, prognosis depends on the underlying and/or accompanying disease(s) but may be similarly poor. For example, recent data on sarcoidosis, which is usually considered a more benign ILD, indicates a considerably higher mortality rate than previously reported [6]. Much remains unknown or debatable for many ILDs because there is a lack of data on the prevalence, incidence, and mortality rates of this group of lung diseases as well as clinical evidence guiding optimal management, healthcare utilisation, and their associated healthcare costs [1].

## 2. Current Epidemiological Estimates for ILDs in the US and across Europe

Most data on the epidemiology of ILDs has been derived from prospective registries of data reported by respiratory physicians. However, there are only a few ILD registries and all have limitations due to the difficulties in accurately diagnosing these conditions; many may not therefore be fully representative of the true populations of ILD patients [1].

One of the first published ILD registries was conducted by Coultas et al. in Bernalillo County, New Mexico, between 1988 and 1990 [7]. According to this registry, the prevalence of ILDs was 20% higher in males (80.9 per 100,000) than in females (67.2 per 100,000). Similarly, the overall incidence of ILDs was slightly more common in males (31.5 per 100,000/year) than females (26.1 per 100,000/year). The authors concluded that the occurrence of ILDs in the general population may be more common than previous estimates based on selected populations, and these disorders may frequently be unrecognised [7].

ILD registries conducted in Europe include a 2004 registry in Greece, which involved a total of 967 cases indicating a prevalence rate of 17.3 per 100,000 inhabitants and an incidence rate of 4.63/100,000 per year [8]. Two Spanish registries also reported a lower incidence of ILDs. One Spanish ILD registry involved 511 cases with an incidence of 7.6 per 100,000/year and a similar male-to-female ratio [9]. A second Spanish registry in the southern provinces involved 744 cases, of which 40.1% of the diagnoses were biopsy confirmed. This reported an annual incidence of 3.62 cases/100,000 (men 4.18 cases/100,000/year; women 3.07 cases/100,000/year) [10].

However, in a more recent study conducted in Turkey between 2007 and 2009 utilising the ATS/ERS consensus criteria from 2002, the incidence of ILD was more similar to that reported in New Mexico, 24.7 per 100,000 person-years for males and 27 per 100,000 person-years for females [7, 11]. This may therefore be a more accurate estimate of the incidence of ILDs because it is also supported by a UK study concerning both IPF and sarcoidosis [12]. This database of UK general practitioners aimed to identify the incidence and mortality rates of IPF and sarcoidosis and was representative of the general population. In this study, the incidence of IPF was 4.6/100,000 per year and 5.0/100,000 per year for sarcoidosis. This study also found a progressive increase in the incidence of IPF between 1991 and 2003, which was not explained by the ageing of the UK population. Since these two relatively predominant presentations—IPF and sarcoidosis—may represent between one-third and one-half of all ILD cases, this would suggest an ILD incidence of 20–30 per 100,000 person-years [1, 12].

These estimated figures are underpinned by a registry conducted in Denmark between 1995 and 2005 involving 21,765 patients with ILDs based on the ICD-8 and ICD-9 classifications identified through the Danish National Patient Registry [13]. This included all in-patients and out-patients with a corresponding hospital discharge diagnosis. The incidence rate fluctuated during the observation period, decreasing from 27.14 per 100,000 person-years to 19.36 per 100,000 person-years. After 1998 the incidence increased considerably, peaking at 34.34 per 100,000 person-years in 2002 and then subsequently decreasing slightly [13].

### 2.1. Epidemiology of Specific ILDs

A number of registries provided relevant information on the epidemiology of specific ILDs, rather than comprehensively, which has been reviewed by Demedts et al. (2001) [14]. For example, in a registry in Belgium involving a total of 362 cases over a 3-year period (1992−1996), the most frequent diagnosis was sarcoidosis (31%), followed by IPF (20%), allergic pneumonia (13%), and vascular collagen diseases (9%) [15, 16]. Similarly, in a single German ILD registry performed between 1995 and 1999 involving 1142 patients, sarcoidosis and IPF were the most frequently registered ILDs [17, 18].

In many of these registries IPF and sarcoidosis were the most common ILDs (Table 1) [19]. However, since most of these studies were conducted before the introduction of the changes in the most recent classifications, the figures concerning ILD as a whole are probably more reliable than those concerning idiopathic interstitial pneumonias specifically [18]. There are also a number of registries dedicated to specific ILDs, such as the INSIGHTS-IPF registry, which focuses solely on IPF [20].

## 3. Rationale for the EXCITING-ILD Registry

As highlighted previously, the only German ILD registry was performed prior to the former and the current ATS/ERS consensus classification [2, 3] and did not reflect a true population-based registry. In this registry only limited data were collected, focusing on the diagnostic procedure, whereas data regarding disease outcome, prognostic factors, pharmacological and nonpharmacological management, and socioeconomic data were not recorded [17, 18]. Similar to Germany, other ILD registries have mainly collected prospective data from specialised ILD outpatient clinics only or used ICD classification or hospital discharge data. Furthermore, data were collected prior to the recent consensus IIP classification in 2013 [1]. Data from community pulmonologists has been lacking in registries conducted to date, which further complicates interpretation of the impact of ILDs in Germany and across Europe. This is important since current data suggests that ILDs may be underestimated and undervalued. For example, symptoms may be misinterpreted as “normal” symptoms of ageing. In addition, the increasing use of CT imaging for nonpulmonary indications (e.g., cardiac or spinal CT) may mean that more subclinical ILDs are being diagnosed than in the past. Altogether, these factors further compound difficulties in ascertaining accurate, population-based epidemiological data on rare, or very rare, diseases such as many of the ILDs.

Registries are increasingly recognised as important observational studies for collecting “real-world” practice information and enabling prolonged follow-up for longer-term data collection. Furthermore, they can be particularly relevant for benchmarking and quality assurance, as individual centres can compare their results with other centres. To address this issue in Germany, the German Centre for Lung Research (DZL) is conducting a prospective ILD registry in association with ambulatory, in-patient, scientific pulmonology organisations, as well as a patient support group. This multicentre, noninterventional, prospective, and observational disease and outcomes ILD registry aims to collect comprehensive and validated data from all healthcare institutions on the incidence, prevalence, regional distribution, characteristics, management, and outcomes regarding all ILD presentations in the real-world setting. Specifically, this registry will collect demographic data, disease-related data such as ILD subtype, treatment locations, diagnostic procedures (e.g., HRCT, surgical lung biopsy), risk factors (e.g., familial ILD), comorbidities, ILD management, analysed by the type and frequency of pharmacological and nonpharmacological interventions, and disease outcomes as well as healthcare resource consumption. The “Exploring Clinical and Epidemiological Characteristics of Interstitial Lung Diseases” (EXCITING-ILD) registry will include in-patient and out-patient ILD healthcare facilities in 3 German states and involve more than 100 sites with a follow-up of at least 5 years. This registry will document comprehensive and current epidemiological data, as well as important health economic data, for ILDs in Germany.

## 4. EXCITING-ILD Registry Objectives

The overall objective of the EXCITING-ILD registry is to generate sociodemographic and medical data relating to ILDs, which will help to answer epidemiological and disease-related questions that will be important in Germany. This registry will also provide an important database for health economic and health services research questions.

Specific objectives of the EXCITING-ILD registry will be to determine

- clinical, disease-related characteristics of ILD patients;
- diagnostic procedures for the identification of ILDs;
- the clinical management of different (sub)types of ILD in real-world practice;
- the outcome of different types of ILDs and of ILD-related therapies from all forms of healthcare facilities.

Main endpoints, reflecting the objectives of this registry, are

- incidence and prevalence of ILD subtypes;
- (disease-related) characteristics of ILD patients;
- diagnostic procedures for the identification of ILDs;
- management of different (sub)types of ILD;
- the outcome of different types of ILDs and of ILD-related therapies measured as time to death;
- ILD management as analysed by the kind and frequency of pharmacological and nonpharmacological therapies;
- prognostic factors in different ILDs;
- lung functional parameters.

Healthcare utilisation will be assessed as a basis for cost estimates for the following healthcare services:

- outpatient treatment;
- hospitalisation due to ILD;
- temporary occupational disability;
- medication;
- additional therapies;
- medical aids and appliances;
- participation in rehabilitation programmes.

Sociodemographic data variables are

- gender;
- year of birth;
- country of birth;
- place of residence;
- profession;
- incapacitated for work;
- health insurance coverage (statutory, private, and granting aid).

## 5. Methods

### 5.1. Study Design and Setting

The EXCITING-ILD registry is a multicentre, noninterventional, prospective, observational disease and outcomes registry across healthcare facilities in Germany. Collected data (retrospective and prospective) will be entered into a web-based system that conforms to the data protection act. The planned duration of the registry is at least 5 years.

### 5.2. Patients and Sites

In the initial 12-month phase it is anticipated that 350 patients will be enrolled and a total of up to 600 patients after the second year. Patients will be recruited in around 100 centres from all forms of healthcare facilities managing ILDs. If comparisons between different ILDs are deemed to be necessary, comparator arms will be defined within the course of the registry, depending on clinical considerations and statistical power.

### 5.3. Inclusion and Exclusion Criteria

The inclusion criteria are adult patients (≥18 years) diagnosed with ILD with signed informed consent and will include the following ILD subtypes (Table 2). Exclusion criteria are patients without ILD.

### 5.4. Study Schedule and Variables

The following data will be collected at baseline and follow-up visits (Table 3). No study-specific patient tests will be performed during this study. Personal data/date of birth/gender will only be recorded once for creation of the anonymous patient identification number.

### 5.5. Patient Withdrawal/Discontinuation

Patient drop-out or withdrawal may occur for the following reasons and will be recorded.

Withdrawal by the investigator due to

- administrative reasons;
- lost for follow-up.

Withdrawal by patient from the study due to

- own request;
- specific request of the sponsor.

In the event that a patient is withdrawn, the registry termination page in the eCRF will be completed, including information on the date of the withdrawal, who initiated the withdrawal, and the reason for withdrawal, if known. Reasonable effort will be made to contact any patient lost to follow-up during the course of the registry to retrieve any outstanding data.

### 5.6. Pharmacological and Nonpharmacological Treatment

The registry will document the management and treatment of patients with ILDs in real-life clinical practice. ILD management will be analysed by the type and frequency/usage of pharmacological and nonpharmacological therapies.

Information on the following pharmacological therapies will be documented at baseline and at follow-up, including any changes in treatment with relevant details on dosing:

- Azathioprine.
- Prednisone.
- NAC (N-acetylcysteine).
- Pirfenidone.
- Nintedanib.
- Cyclophosphamide.
- Methotrexate (MTX).
- Mycophenolate mofetil (MMF).
- Rituximab.
- Inhaled antiobstructive therapy (LAMA, LABA).
- Inhaled corticosteroids (ICS).
- Rapamycin/sirolimus.
- TNF alpha blocker.
- Clinical trial medication.
- Other treatment.

Information on the following nonpharmacological treatments will be documented:

- Physiotherapy or treatment of other allied healthcare professionals.
- Long-term oxygen therapy.
- Noninvasive ventilation.
- Lung transplantation or listed for lung transplantation.
- Patient support group.
- Participation in rehabilitation programmes.
- Others.

### 5.7. Statistical Methods

A Statistical Analysis Plan (SAP) has been developed prior to initiation of the registry and final analysis of the trial will be performed by syneed medidata GmbH. All observational data collected will be analysed descriptively using established statistical and epidemiological methods. Time to death will be analysed by means of Kaplan-Meier survival analysis. In addition, further secondary inferential methods may be specified in the SAP if deemed appropriate. If not specified otherwise in the SAP, missing data will not be imputed. Subjects with incomplete data will be included in the analysis and for each variable; the number of subjects with missing data will be reported. Interim analyses comprising descriptive summaries of the data collected in the CRF will be produced every 3 months.

*Sample Size*. In the initial phase of 12 months, enrolment of up to 350 patients is anticipated. At the end of year 2, it is anticipated that up to 600 patients will be enrolled. This sample size (*n* = 350) allows for a log-rank test at a fixed time given a hazard ratio ≤0.68 at alpha = 0.05 with a power of 95%. Equally, this allows for estimating an event with 50% frequency at a confidence interval of ±5% at a 95% confidence level. All subjects with at least one documented postbaseline visit will be entered into the full analysis set (FAS). Further analysis sets may be defined in the SAP.

### 5.8. Data Collection

Participating physicians will enter the data of enrolled patients directly in a standardised, web-based electronic Case Record Form (eCRF). All patient data collected will be entered into the database.

### 5.9. Adverse Event Reporting

In this disease and outcomes registry, no special adverse drug reporting requirements apply. The physician should report any adverse drug reactions, according to his/her routine methods, as regular spontaneous reports in accordance with Volume 9A of The Rules Governing Medicinal Products in the EC as well as in accordance with local laws and regulations.

### 5.10. Monitoring

In accordance with ICH-GCP Guidelines, selective monitoring visits will be performed during the course of the study. These visits will include checking adherence to the protocol, the completeness and accuracy of the data, patient confidentiality, GCP guidelines, and national laws. Source data verification will be used for assessment for complete and reliable documentation. Monitors will have direct access to all source data, including electronic medical records, and/or documents in order to facilitate data verification. An Advisory Board will monitor the collected data on a regular basis and provide advice on all scientific issues, including the dissemination plan. Representatives of a patient support organisation are also included in the Advisory Board.

### 5.11. Ethical Approvals

The Ethics Committee of the Medical Faculty of the University of Heidelberg, Germany, approved the study protocol, the data collection forms, and the patient information forms. All participating centres will obtain approval from their local ethics committees. The registry will be conducted in accordance with ethical principles founded in the Declaration of Helsinki.

## 6. Methodological Considerations and Limitations

The EXCITING-ILD registry is prospective and recruits consecutive patients, thus limiting selection bias. The main limitations of this study are those inherent to any registry. Given that this is an observational, nonrandomised study, no causal associations may be derived. Clinical decisions of the treating physicians including the final diagnosis of the respective ILD and decisions that may assign patients to different drugs based on disease severity, disease duration, presence of comorbidities, and other factors can potentially introduce allocation or channelling bias and confound the association between treatment and outcomes. Particularly consider that patients will be included from centers with different levels of expertise in ILD. Also, other relevant aspects such as more information on 6 MWD, types of assessment of pulmonary hypertension, BAL, and TBLB data will therefore not be assessed. Yet, as registries are aimed at reflecting the “real-world” situation of diagnosis and treatment of the respective diseases, also in our registry these limitations mirror the current care of patients with interstitial lung diseases and are therefore of high value.

National epidemiological data on the true incidence and prevalence of ILDs will not be derived, but the registry will provide representative data on the situation of expert centres, large pulmonary hospitals (referral centres) in which many ILD patients are treated, as well as patients treated by general pulmonologists in community practices. On the basis of these data after final recruitment of all centers and in potential future collaboration with health insurances, we will try to assess incidences and prevalence of ILDs in these regions to reflect the German situation more properly.

## 7. Dissemination of Information and Publications

EXCITING-ILD will be registered at ClinicalTrials.gov. A study report will be written upon completion with results to be reported on ClinicalTrials.gov and as peer-reviewed publications.

## 8. Conclusions

Over the past year we have witnessed significant progress in the clinical evaluation and management of ILDs. Advances have been made in the elucidation of pathobiological mechanisms of several forms of ILDs and new therapeutic options have been introduced for various forms of ILDs, such as pirfenidone and nintedanib in IPF [21, 22] and sirolimus in LAM, just to mention a few [23]. Despite these developments, however, ILDs still represent a group of serious, life-threatening conditions associated with high mortality [4]. ILDs are therefore of high interest for the healthcare system as well as for payers when considering costs and the burden of disease. However, data on economic issues related to the care and management of ILDs is limited, with most information to date focussing mainly on IPF only [24, 25]. There is, therefore, a pressing need for assessing the cost-effectiveness of interventions such as pharmacotherapy but also nonpharmacological therapies across the other ILDs.

In this context, registries can help to inform relevant stakeholders about particular diseases and population behaviour patterns, as well as associations with disease development and how to improve and monitor the quality of healthcare [26]. With these objectives the EXCITING-ILD registry aims to provide guidance on addressing unmet needs in caring for ILD patients. To our knowledge the EXCITING-ILD registry will be the first registry to document comprehensive and current epidemiological data as well as important health economic data for ILDs.

## Figures and Tables

**Table 1:** Comparison of the distribution of interstitial lung diseases (ILDs) in respiratory physicians' prospective registries [18].

	Flanders (Belgium) (1992–1996)		Germany (1995)	Italy (1997–1999)	Spain/RENIA (1998–2000)	Spain/SEPAR (2000-2001)	Greece (2004)	
	Prevalent cases	Incident cases	Incident cases	Prevalent cases	Incident cases	Incident cases	Prevalent cases	Incident cases
Subjects (*n*)	362	264	234	1138	744	511	967	254
Idiopathic								
Sarcoidosis	112 (31)	69 (26)	83 (35)	344 (30)	87 (12)	76 (15)	330 (34)	60 (23)
IPF/IIP	62 (17)	50 (19)	76 (32)	417 (37)	287 (39)	215 (42)	234 (24)	66 (25)
COP/BOOP	10 (2.3)	9 (3.4)	16 (6.8)	57 (5.0)	38 (5.1)	53 (10)	51 (5.3)	18 (7.0)
(C)EP	9 (2.2)	7 (2.7)		27 (2.3)	—	—	21 (2.2)	7 (2.7)
CTD	27 (7.5)	19 (7.2)	5 (2.1)	—	69 (9.3)	51 (19)	120 (12)	30 (12)
Vasculitis^*∗*^	5 (1.4)	4 (1.5)	2 (0.8)	25 (2.2)	—	—	14 (1.5)	6 (2.3)
EG/HX	13 (3.6)	7 (2.7)	—	73 (7.2)	6 (0.8)	15 (3)	37 (3.8)	7 (2.7)
Nonidiopathic								
EAA	47 (13)	32 (12)	25 (11)	50 (4.3)	38 (5.1)	34 (7)	25 (2.6)	7 (2.7)
Drug^†^	12 (3.3)	12 (5)	6 (2.6)	21 (1.8)		21 (4)	17 (1.8)	4 (1.5)
Pneumoconiosis^‡^	19 (5.0)	18 (6.8)	6 (2.6)	—	55 (7.4)	—	20 (2.0)	8 (3.1)
Variable aetiology								
Nonspecific fibrosis	33 (9.1)	27 (10)	12 (5.1)	—	69 (9.3)	—	82 (8.5)	40 (15)
Others	13 (3.8)	10 (3.8)	—	124 (11)	76 (10)	9 (2)	15 (1.5)	6 (2.3)

Data are presented as *n* (%), unless otherwise stated. RENIA: Registry of Interstitial Pneumopathies of Andalusia; SEPAR: Sociedad Española de Neumología y Cirugía Torácica; IPF: idiopathic pulmonary fibrosis; IIP: idiopathic interstitial pneumonia; COP: cryptogenic organising pneumonia; BOOP: bronchiolitis obliterans organising pneumonia (not necessarily cryptogenic); (C)EP: (chronic) eosinophilic pneumonia; CTD: connective tissue disease; EG/HX: eosinophilic granuloma/histiocytosis X; EAA: extrinsic allergic alveolitis (hypersensitivity pneumonitis).

^*∗*^Goodpasture's, granulomatosis with polyangiitis (Wegener's), Churg-Strauss, and so forth.

^†^Radiation was also included in the Italian and SEPAR registries.

^‡^Coal worker's pneumoconiosis was excluded in the Flemish, Italian, and SEPAR registries.

Adapted from the European Lung White Book Chapter 22 [18].

**Table 2:** ILD patients and subtypes^*∗*^ eligible for enrolment in the EXCITING-ILD registry.

Idiopathic interstitial pneumonias (IIPs)	Idiopathic pulmonary fibrosis (IPF) Idiopathic nonspecific interstitial pneumonia (NSIP) Desquamative interstitial pneumonia (DIP) Respiratory bronchiolitis-associated interstitial lung disease (RB-ILD) Cryptogenic organising pneumonia (COP) Idiopathic lymphoid interstitial pneumonia (LIP) Acute interstitial pneumonia (AIP) Rare forms of IIPs (e.g., pleuropulmonary fibroelastosis) Nonclassifiable IIP

Granulomatous lung disease	Sarcoidosis Berylliosis Other (e.g., involvement in chronic inflammatory liver and gut diseases, except EAA)

Hypersensitivity pneumonitis (extrinsic allergic alveolitis (EAA))	Farmer's lung Bird keepers' lung disease Origin unknown Other

Rheumatic and connective tissue diseases with pulmonary involvement such as	Connective tissue disease (subtype) Vasculitis Rheumatoid arthritis

Pneumoconiosis	Asbestosis Silicosis Other

Other forms	Pulmonary lymphangioleiomyomatosis Pulmonary Langerhans' cell histiocytosis Pulmonary alveolar proteinosis Eosinophilic pneumonia Post-ARDS fibrosis

Drug-related	

Radiotherapy associated	

Fibrosis in emphysema patients without signs of other ILDs (CPFE)	

Others	

Not classifiable	

^*∗*^For each subtype it will be queried whether or not a diagnosis of concomitant emphysema in ILD was made.

**Table 3:** List of variables to be documented (if available) at baseline and scheduled visits.

	Baseline	Follow-up every 6 months
*Eligibility criteria*	*✕*	

*Demographic/sociodemographic data*	*✕*	
Gender, country of birth, place of residence with zip code, profession, year of birth, and date of ILD first diagnosis if available	*✕*	
Weight and height, BMI	*✕*	*✕*
Incapacitated for work caused by ILD	*✕*	*✕*
Health insurance coverage	*✕*	*✕*

*Risk factors*	*✕*	
Smoking status, profession, familial ILD, HIV		

*Profession*	*✕*	*✕*
Profession, in work, unemployed, student, retired, other, incapacitated for work caused by ILD, disease-related absent days past six months		

*Comorbidities with prognostic impact*	*✕*	*✕*
Gastroesophageal reflux (treatment), pulmonary hypertension (treatment), emphysema		

*Disease-related data*		
Subtype of the ILD, date of diagnosis, multidisciplinary diagnosed (e.g., ILD board), onset of first symptoms	*✕*	
Surgical lung biopsy	*✕*	

*CT*		
CT scan also analysing whether HRCT (thin-section CT, thin-slice spiral CT, <2 mm thickness) was performed	*✕*	*✕*

*Lung function data*		
(VC_max_, FVC, FEV_1_, FEV_1_/FVC, DLCO-SB, DLCO-VA, TLC) at time of diagnosis and current values	*✕*	*✕*
6-minute walking test (6-MWT) distance at time of diagnosis and current values	*✕*	*✕*

*Previous and current therapy of ILD*	*✕*	*✕*
(details and dosage)		

*Other therapies*	*✕*	*✕*
Physiotherapy or treatment from other allied healthcare professionals, long-term oxygen therapy, noninvasive ventilation, lung transplantation or listed for lung transplantation, patient support group, participation in rehabilitation programmes, others		

*Hospitalisation*	*✕*	*✕*
Caused by ILD or ILD-associated during the last 6 months		
Not caused by ILD		

*Out-patient clinic*	*✕*	*✕*
Was the patient seen during the last 6 months by the reporting physician/by additional physicians		

*Medical aids and appliances*	*✕*	*✕*
Vaporiser, aids for elimination of secretions, other due to ILD (e.g., wheelchair)		
